# Supplementary material for: Low P66shc with High SerpinB3 Levels Favors Necroptosis and Better Survival in Hepatocellular Carcinoma
Source: Biology (Basel). 2021 Apr 23;10(5):363. doi: 10.3390/biology10050363 (PMC8145214; doi:10.3390/biology10050363)
Supplement: Supplementary file 1 [file biology-10-00363-s001.zip › Supplementary files/Biology_Supplemental Tables.pdf]

Supplemental Table 1. Clinical and histological characteristics of patients included in the study.

**No. of patients**: 67	
**Age** (mean years ± SD)	62,9 ± 9,5
**Male sex** (%)	50/67 (74,62%)
**Etiology**	
• HBV infection (%)	10/67 (14,92%)
• HCV infection (%)	28/67 (41,8%)
• Alcohol use (%)	26/67 (38.8%)
• Other . (%)	11/67 (11.67%)
**Pathology**	
• Number of nodules . (min-max)	1,46 (1-9)
• Nodule diameter media – mm (min-max)	53,6 (13-190)
• Vascular invasion	36 (53.73%)
- Microscopic (%)	31 (46.26%
- Macroscopic (%)	5 (7.46%)
**Grading**	
• GI . (%)	11 (16.41 %)
• GII (%)	28 (16.41%)
• GIII . (%)	25 (37.3%)
**Median survival** - months (min-max)	22,1 (1,333-97,43)

Supplemental Table 2. Nucleodite sequences of the primers used in the study.

Gene name	Species	Primer sequences
p66shc	Homo Sapiens	Fw 5’- GAAGGAGCACAGGGTAGTGG-3’<br>Rv 5’- CCACTCCGGAATGAGTCTCT-3’
SerpinB3	Homo Sapiens	Fw 5’-GCA AAT GCT CCA GAA GAA AG-3’<br>Rv 5’-CGA GGC AAA ATGAAAA AGA TG-3’
β-Catenin	Homo Sapiens	Fw 5'-TGGTGCCCAGGGAGAACCCC-3‘<br>Rv 5'-TGTCACCTGGAGGCAGCCCA-3'
TGF-β	Homo Sapiens	Fw 5'-AAGTGGACATCAACGGGTTC-3‘<br>Rv 5'-GTCCTTGCGGAAGTCAATGT-3'
IL-6	Homo Sapiens	Fw 5’-AGTTGCCTTCTTGGGACTGA-3’<br>Rv 5’-CAGAATTGCCATTGCACAAC -3’
IL-10	Homo Sapiens	Fw 5’-CCAAGCCTTATCGGAAATGA-3’<br>Rv 5’- TTTTCACAGGGGAGAAATCG -3’
TNF-a	Homo Sapiens	Fw 5’-AGCCCCCAGTCTGTATCCTT-3’<br>Rv 5’- CTCCCTTTGCAG AACTCAGG -3’
β-actin	Homo Sapiens	Fw 5’- AGCCATGTACGTAGCCAGAA-3’<br>Rv 5’- CTCTCAGCTGTGGTGGTGAA-3’
β-Catenin	Mus Musculus	Fw 5’-'TACGAGCACATCAGGACACC-3‘<br>Rv 5'-AATCCGGTTGTGAACGTCCC-3'

Fw= forward; Rv= revers
