# Supplementary material for: Low P66shc with High SerpinB3 Levels Favors Necroptosis and Better Survival in Hepatocellular Carcinoma
Source: Biology (Basel). 2021 Apr 23;10(5):363. doi: 10.3390/biology10050363 (PMC8145214; doi:10.3390/biology10050363)
Supplement: Supplementary file 1 [file biology-10-00363-s001.zip › Supplementary files/Fasolato et al. Supplemental Figures Rev.pdf]

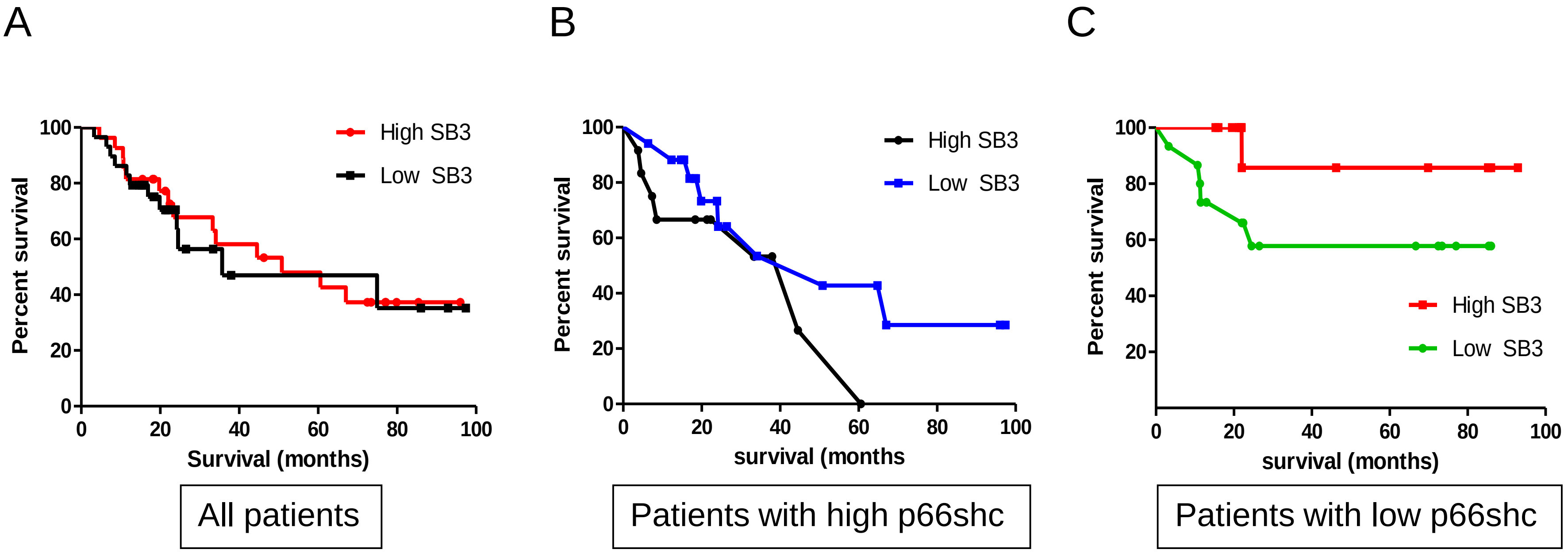

Supplementary Figure 1. Survival curves in relation to SerpinB3 and p66shc expression. A) Kaplan-Meier survival curves in the cohort of 67 patients with hepatocellular carcinoma, divided in two groups according to high (≥ median value) or low (< median value) SerpinB3 (SB3) mRNA expression level (p= ns); B) Kaplan-Meier survival curves of HCC patients with high expression of p66shc, divided in two subgroups with high or low expression of SB3 (p = ns); C) Kaplan-Meier survival curves in HCC patients with low expression of p66shc, divided in two subgroups with high or low expression of SB3 (p = NS).

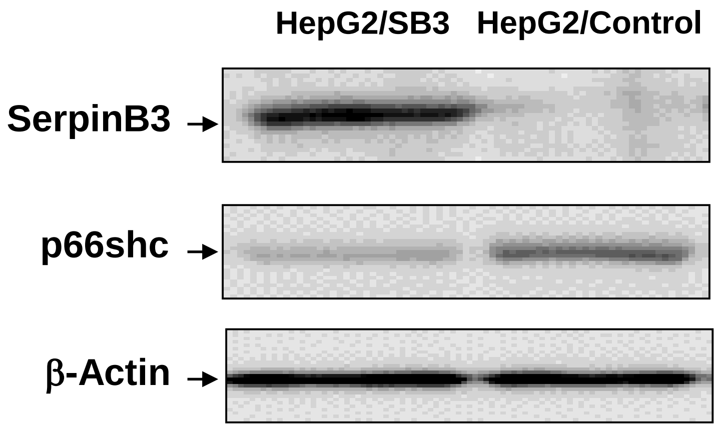

Supplementary Figure 2. Representative Western blot for SERPINB3 and p66shc in HepG2 clone3 cells overexpressing SERPINB3, (Hep2/SB3) and in control HepG2 cells, transfected with the plasmid vector alone (HepG2/Control).

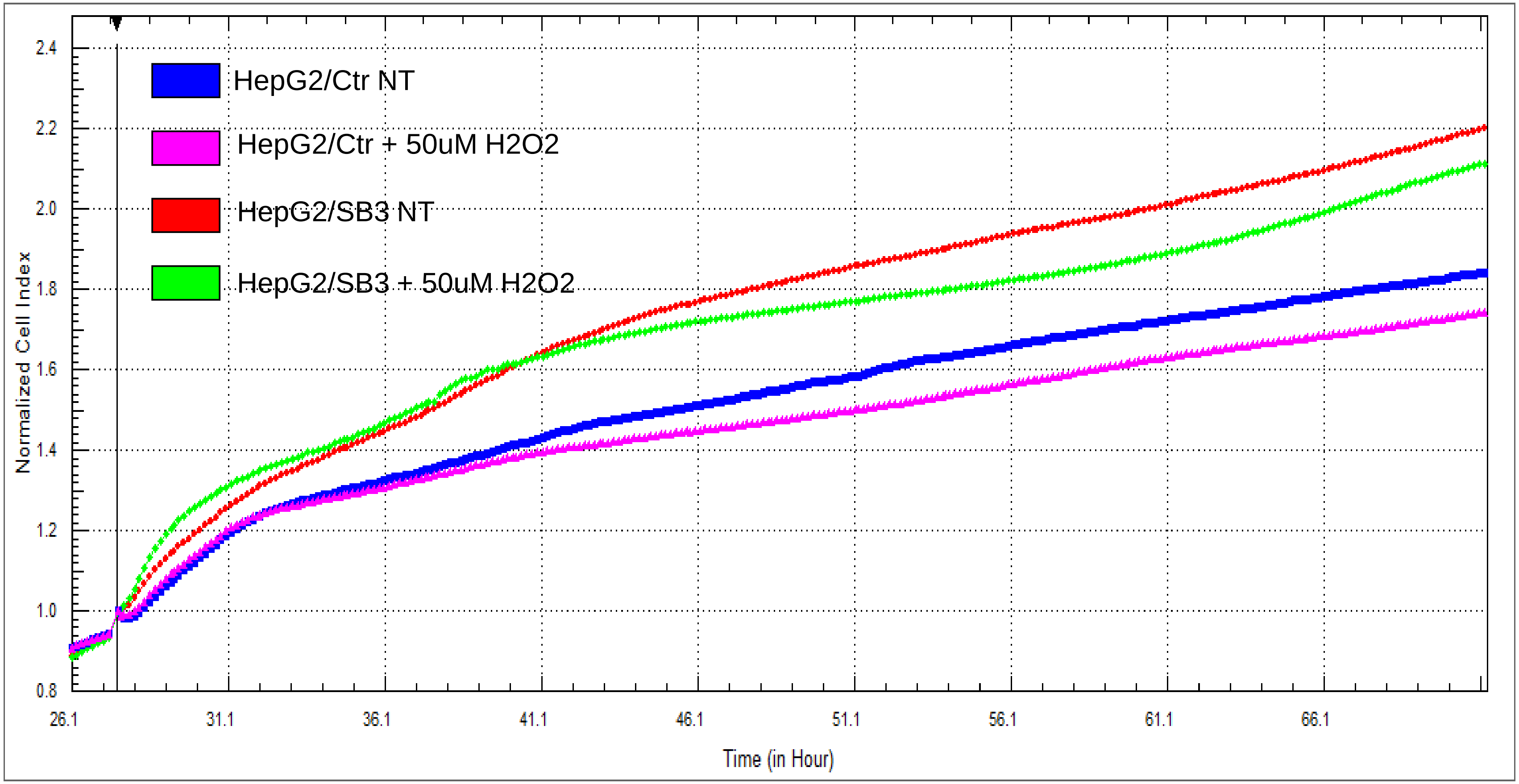

Supplementary Figure 3. Real time proliferation of HepG2 cell overexpresing SerpinB3 (HepG2/SB3) and of their relative controls (HepG2/Ctr) in presence or absence of low H_2O_2 concentration (50 mM).

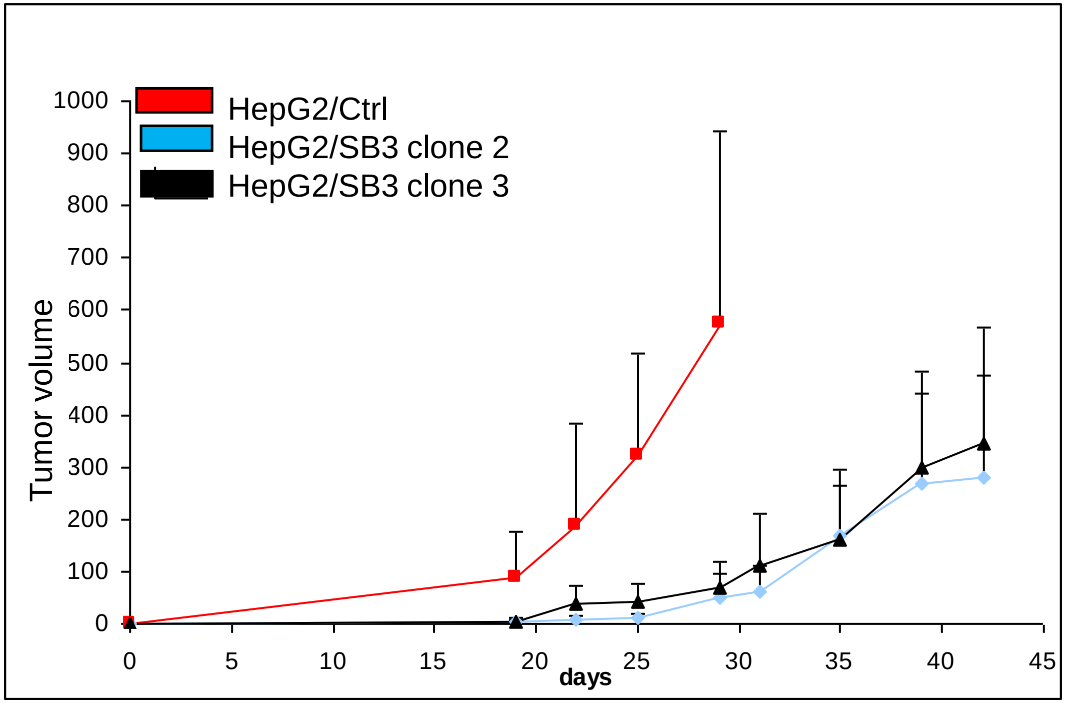

Supplementary Figure 4. Tumor growth in Rag-c57 mice (N.4 for each group) inoculated with control HepG2 cells (HepG2/Ctrl, Red line) or with two different clones overexpressing SerpinB3 (HepG2/SB3 clone 2, blu line and HepG2/SB3 clone 3, black line). Each point represent mean tumor volume and bars represent SD.

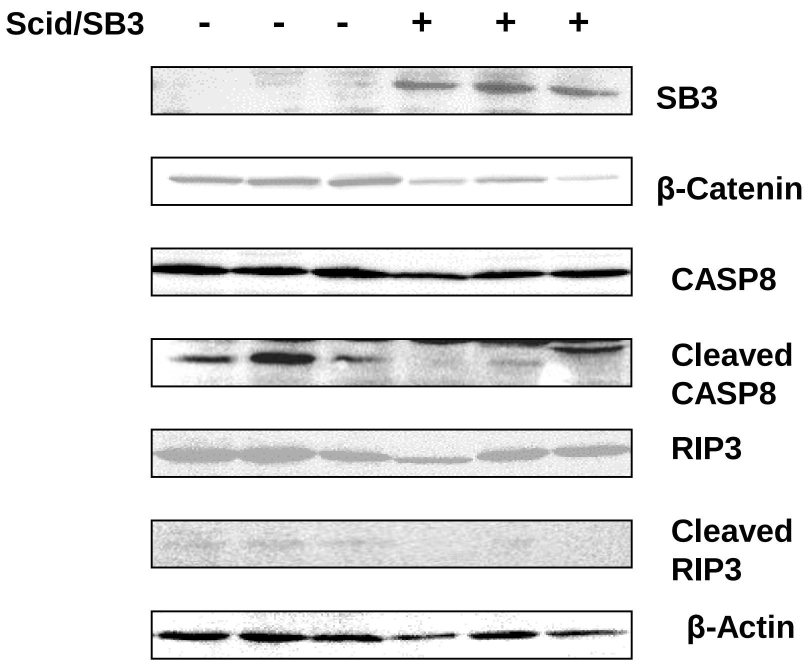

Supplementary Figure 5: Representative example of Western blot of SerpinB3 (SB3), β-Catenin, Caspase 8 (CASP8), Cleaved CASP8, RIP3, Cleaved RIP3 and of the housekeeping β-Actin proteins in tumors of 6 Scid mice inoculated with control HepG2 cells (Scid/SB3 -) or with HepG2 cells overexpressing SerpinB3 (Scid/SB3 +).
